# Stemness-driven clusters in ovarian cancer: immune characteristics and prognostic implications

**DOI:** 10.3389/fonc.2025.1577283

**Published:** 2025-06-11

**Authors:** Xinyan Zeng, Wentian Wu, Xiaoqin Li, Xiaorui Wu, Yingying Du, Ping Li

**Affiliations:** ^1^ Department of Chinese Integrative Medicine Oncology, The First Affiliated Hospital of Anhui Medical University, Hefei, China; ^2^ Department of Integrated Traditional Chinese and Western Medicine, Anhui Medical University, Hefei, China; ^3^ Department of Medical Oncology, The First Affiliated Hospital of Anhui Medical University, Hefei, China; ^4^ Graduate School of Anhui University of Traditional Chinese Medicine, Hefei, China

**Keywords:** ovarian cancer, stemness, immune checkpoints, drug resistance, tumor organoid

## Abstract

**Background:**

Ovarian cancer (OC) is the most common malignant gynecological tumor. Cancer cells with high stemness often exhibit resistance to anti-tumor therapies, contributing to recurrence and poor prognosis. However, stemness-related subtypes in OC and their therapeutic implications remain underexplored.

**Methods:**

We identified stemness-associated genes by comparing transcriptome profiles between adherent and sphere-forming SKOV3 cells. Unsupervised clustering was applied to define stemness-related molecular subgroups in OC patients. A prognostic model was constructed using WGCNA and LASSO regression, and a nomogram was developed by integrating clinicopathological variables. Differences in the tumor microenvironment (TME), tumor mutation burden (TMB), immune checkpoint expression, and drug sensitivities were evaluated between risk groups. Single-cell RNA sequencing was used to investigate stemness-related cell types. Functional assays were conducted to validate the role of AKAP12 in OC progression.

**Results:**

Three distinct stemness-related subgroups were identified with significant differences in prognosis and immunological features. Fibroblasts were identified as major contributors to the maintenance of stemness traits in the TME. AKAP12 was found to be positively associated with stemness phenotypes. Knockdown of AKAP12 reduced tumor sphere formation, impaired cell migration, and enhanced cisplatin sensitivity. Immunohistochemistry in clinical samples and OC organoids confirmed the correlation between AKAP12 and the immune checkpoint molecule OX40L.

**Conclusion:**

This study establishes a novel stemness-related gene signature for prognosis prediction and therapeutic stratification in OC. AKAP12 was identified as a potential biomarker and therapeutic target, offering new avenues for precision treatment in stemness-driven OC.

## 1 Introduction

As per epidemiological statistics, ovarian cancer (OC) is the eighth common cancer in terms of female prevalence and remained to be the most fatal gynecologic cancer in 2020 (1, 2). The in-depth dissections of single-cell methodology illustrate substantial heterogeneity of OC, underscoring the phenomena of chemoresistance, immune-suppression, angiogenesis, and distant migration that can be result in (3, 4). Additionally, distinct subtypes within sophisticated and varied TME are observed in independent OC tissues changing the notion of treating OC as a single homogeneous entity (5). Furthermore, the number of viable approaches will come to a failure, leading to a decline of overall survival (OS), when drug resistance emerges, since the majority patients encounter resistance and therapeutic failure (6–8). A meta-analysis indicates immunotherapeutic agents are regarded as possessing high levels of safety and efficacy (9). Drawing upon that specific cell type expressing molecular characteristics and potential therapeutic targets, exerts profound impact on the therapeutic responses and OS (10, 11), it’s advisable for us to explore and investigate novel biomarkers to guide personalized treatment strategies.

On reviewing previous studies, we noticed that the mechanisms leading to OC drug resistance include DNA damage repair, alterations in transmembrane transport, and abnormal signaling pathways. Cancer stem cells are important components that have not yet been fully explored (12–14). Based on the postulated cancer stem cell theory, OC stem cells maintain tumorigenesis at the metastatic site of the tumor, and a subpopulation of cancer stem cells possesses metastasis and drug resistance phenotypes (15). As a rare subgroup of cancer cells with the capacity for chemotherapy resistance, OC stem cells have evolved from tumor cells that have the capacity to invade and resist chemotherapy (16). On the other hand, the widespread application of conventional chemotherapy has been linked to the upregulation of stem characteristics and facilitation of epithelial to mesenchymal transition and exacerbation metastasis, thereby intensifying the metastatic potential of clinical treatment (17, 18). The intricate interference between OC stem cells and the immune and non-immune components lead to the dysfunction of immune surveillance and suppression, creating a favorable environment for the transplant and proliferation of stem cells (19, 20). Therefore, the investigation of TME can present a promising avenue for seeking novel therapeutic targets.

Previous studies have evaluated the potential role of immune checkpoints in the stem cell subgroups of OC cells and found strong bonds, which elucidated the feasibility of immune checkpoint inhibitor therapy (21, 22). Crosstalk exists between cancer stem cells and immune cells. Cancer stem cells can inhibit the transport, maturation, and differentiation of antigen-presenting cells (APCs), such as dendritic cells (DCs) and macrophages. Suppressor immune cells can be recruited using cytokines or chemokines secreted by stem cells (23). Therefore, they contribute to the formation of an immunosuppressive microenvironment and facilitate the escape of cancer stem cells from immune surveillance. Notably, inflammation arising from cytokines secreted by tumor-associated immune cells can lead to the migration of cancer stem cells to maintain tumor growth (24, 25). In addition, given that cancer stem cells interact with immune checkpoint molecules to produce immunosuppressive factors, exploring and targeting novel immune checkpoints may be a potentially valuable approach for individual treatment (26, 27).

In recent years, traditional transcriptome analysis has been used to detect the differential expression of genes in multiple samples, ignoring the crosstalk between cellular compositions. Currently, single-cell RNA-sequencing (scRNA-seq) analysis has made a breakthrough in identifying unique cells with a high revolution and revealing interactions among different subtypes (28–30). Therefore, we first identified stem cell genes by analyzing transcriptome sequencing data before and after OC cell differentiation. To evaluate the performance, unsupervised clustering was employed to identify specific clusters with high levels of stemness. By applying a weighted gene correlation network analysis (WGCNA), we filtered out genes that were strongly correlated with stemness. Subsequently, risk genes were identified to construct a prognostic model, and their coefficients were calculated using machine learning algorithms. We explored its correlation with immune infiltration, molecular function, and drug resistance to identify possible therapeutic targets. Finally, by integrating multiple single-cell datasets, signaling pathways regulating the formation of stem cells and correlations among all cell types in different malignant groups were discovered. Though large amounts of studies reckoned the key role of stem cells in relapse and resistance, the approach to eradicate the cancer stem cells hadn’t developed yet (31).

This study contributes to the identification of therapeutic targets for individual treatments by investigating the prognostic value of stem cell genes. The established risk model may robustly predict the survival rate and response to immunotherapy.

## 2 Materials and methods

### 2.1 Datasets acquisition and preprocessing

Firstly, the clinical follow-up information, expression data and somatic mutation data of 379 OC patients in TCGA database could be obtained from the GDC hub of UCSC Xena browser (https://xenabrowser.net/). Due to the inconsistence of gene length, we transformed the original expression data to the transcripts per million (TPM) values and conducted the exponential transformation of data for superior comparability. Meanwhile, patients’ microarray data and survival information (GSE26712) (32), (GSE32062) (33) and gene expression data of SKOV3 cells in different status (GSE232783) (34) could be obtained from GEO database (https://www.ncbi.nlm.nih.gov/geo/). Under stringent quality control, scRNA-seq data of primary OC samples covering high- and low-grade was acquired as well (35). Furthermore, in order to eliminate the potential cross-dataset batch effects, we employed “sva” package during the analyses based on the empirical Bayes framework (36).

### 2.2 Function enrichment analyses of differentially expressed genes

Building upon the result detected in prior research (34), it’s evident that the emerge of aggresomes not only preserves the stem-like properties, but also remarkably strengthens the aggressiveness of OC cells. Therefore, by meticulously comparing the hub genes altered during the transition to aggresomes, we aim to seek and discern novel stem genes, which are anticipate in exerting fatal impact on OC patients’ survival outcomes. In order to identify differentially expressed genes (DEGs), we utilized “limma” R package (37) to filter genes with significant changes before and after OC cells differentiation. Our selection was under the threshold of adjusted *p* value < 0.05 and |log2foldchange| ≥ 1. To investigate the molecular mechanisms, biological functions and cellular components of stem genes, we performed gene ontology (GO) and Kyoto Encyclopedia of Gene and Genome (KEGG) enrichment analysis via gene datasets acquired from “clusterProfiler” package (38). Taking interaction among genes into consideration, we mapped a line diagram to manifest the alteration of various signaling pathways by conducting Gene Set Enrichment Analysis (GSEA) (39). The criteria of selection were set as *p* < 0.05 and false discovery rate (FDR) < 0.25.

### 2.3 Consensus clustering of stem genes in OC

After conducting univariable Cox regression analysis to select stem genes with prognostic value, we further utilized “ConsensusClusterPlus” R program (40) to apply unsupervised clustering of stemness characteristics for patients in TCGA cohort. By separating all the patients into k (k=2-9) subgroups, we determined the favorable cluster numbers and the expression profile of stem genes was calculated to ensure the robust differences among subgroups. Meanwhile, Kaplan-Meier (KM) curves were represented to investigate whether OS exhibited difference in all subgroups.

### 2.4 Comparing the immune infiltration between stem subgroups

For detecting the correlation between stem genes and immune infiltration, “estimate” R package (41) was applied to calculate the immune score, estimate score, stromal score and tumor purity with estimate algorithm. Subsequently, we employed single-sample gene set enrichment (ssGSEA) algorithm (42) to explore the abundance and distribution of 28 immune cells in stem subgroups. Through analyzing the expression of immune checkpoints and evaluating the immune infiltration extent, we would successfully seek for the association between immune functions and stem genes.

### 2.5 Phenotype score and WGCNA

To estimate the stem level of each sample, we conducted ssGSEA algorithm, which could unfold the coordinated upward or downward adjustments of imported gene sets utilizing “GSVA” R package (43). Through calculating stem score of OC samples in TCGA cohort, we acknowledged the enrichment degree of stem genes. What’s more, WGCNA could divide high-throughput data into multiple co-expression modules and illustrated the potential linkage between modular genes and clinical phenotypes (44–46). For seeking genes with significant biological and clinical traits, it’s advisable for us to select related modules, whose genes could be natural candidates for further analysis. Genes with the same pattern were separated into the same module and associations between modules and clinical traits were assessed using Pearson’s correlation analysis.

### 2.6 Construction and validation of stem prognostic model

In the first place, univariable Cox regression analysis was performed to choose stem genes with prognostic value brought from the previous step. The least absolute shrinkage and selection operator (LASSO) Cox regression had a capacity of regularization and reducing data to dimensionality when “glmnet” R package (47) was invoked. When applied to the construction of the model, LASSO Cox regression would narrow down the number of stem genes and determine the optimal composition of risk signature with the penalty parameter (λ) identified by minimum criteria. With this approach, we could avoid the overfitting of the model and enhance the accuracy of prediction. Subsequently, the risk score for each OC patient was calculated according to the following formula:


$$\text{risk score}=\sum_{\text{i}=1}^{\text{n}}\left({\text{coef}}_{i}{\;*\text{Exp}}_{i}\right)$$

(coef_i_: the coefficient value, Exp_i_: the expression value of the prognostic genes) Serving the best cut-off value of risk score as the threshold value, patients with OC were separated into high- and low-risk groups. “survminer” package (48) was used to display survival curves in two risk subgroups. Taking visibility of distinctiveness into consideration, we applied principal component analysis (PCA) (49) to demonstrate the compositions of the data. Finally, the area under the curve (AUC) was calculated with “survivalROC” program (50) to validate the model’s capability of predicting the 3- and 5-year OS. Two independent datasets, GEO cohorts (GSE26712, GSE32062) were utilized as validation sets to verify the generalizability of the model.

### 2.7 Establishment of a nomogram

To assess prognostic features of all patients, a nomogram was constructed involving risk score value and clinicopathological parameters using “rms” (51). By integrating prognostic factors, the nomogram could evaluate the outcomes between high- and low-risk patients with favorable efficacy. Subsequently, calibration curve and Decision Curve Analysis (DCA) curve were depicted to measure the difference between predictive and actual values and evaluate the net clinical benefit of each independent index.

### 2.8 Immune infiltration and tumor mutation burden analysis

With the application of ssGSEA as mentioned above, the proportions of 28 immune cells and the expression of immune checkpoints could be calculated and the different between high- and low- risk subgroups could be distinguished by Wilcoxon rank-sum test. Equally, the differential immune signaling pathways were investigated by “GSVA” R package. Tumor burden (TMB), which represents the number of mutations per megabase (Mut/Mb) of DNA that is sequenced in malignant tumor could be a candidate prognostic factor and was applied to clinic widely (52, 53). Considering TMB as a candidate biomarker, we discussed its difference in adjective risk subgroups and revealed its prognostic value by KM analysis.

### 2.9 Drug sensitivity

Based on “oncopredict” R package (54), we could download GDSC and CTRP data matrixes, which were used as training cohorts to predict the drug response. Serving the area under the dose-response curve values as basis, we calculated the IC50 value (half-maximal inhibitory concentration) to detect the response of patients with OC to common drugs. Chemotherapy or immunotherapy drugs exhibiting with *p* < 0.05 in different risk clusters were selected as candidate drugs for further analysis.

### 2.10 Analysis of single-cell sequencing data

To process the raw single cell data, we utilized “Seurat” R package (55) to build a new Seurat object. An initial quality control process was implemented to filter a series of high-quality cells, which encompass 400–7000 RNA features and possess a mitochondrial gene proportion of less than 5%. Simultaneously, genes that express within less than three cells are also exclude to ensure the concentration on biologically relevant factors. Moreover, “LogNormalize” function is employed to normalize and adjust features across distinct cells, thereby facilitating their comparability. Following the identification of hypervariable genes, Principal Component Analysis (PCA) is conducted to reduce the dimensionality of high-throughput data and select 15 principal components (PCs) to capture major variants, as determined by the ElbowPlot function. Finally, the cell clusters were generated by FindClusters algorithm with the revolution of 0.5 and were presented with Uniform Manifold Approximation and Projection (UMAP), which had excellent performance and good scalability. The cell type annotation was completed by manual operation to ensure the accuracy of results. The “decoupleR” (56) function was also applied to evaluate the strength of multiple signals in all cell clusters.

### 2.11 Cell-cell communication analysis

We conducted the CellChat R package (57) to manifest the number and intensity of intercellular interactions. Furthermore, to investigate the abnormal signaling pathways and their stability, we performed the pattern recognition function of CellChat to evaluate the strength of incoming and outcoming signaling pathways. Meanwhile, the heatmap was displayed to clarify the contribution of each cell cluster in diverse pathways.

### 2.12 Cell culture and tumor sphere-formation assay

SKOV3 cells were cultured in McCoy’s 5A medium supplemented with 10% fetal bovine serum (FBS) at 37°C in a humidified atmosphere with 5% CO_2_. Tumor sphere-formation assay was performed to evaluate the stemness characteristics of SKOV3 cells. Single-cell suspensions were prepared and seeded into ultra-low attachment 96-well plates (Corning) at a density of 1000 cells per well. After seeding, the plates were centrifuged at 1500 rpm for 10 minutes to facilitate cell aggregation at the bottom of each well. Cells were cultured in serum-free DMEM/F12 medium (Gibco) supplemented with 1× B27 supplement (Gibco), epidermal growth factor (EGF, 20 ng/mL, MCE), and basic fibroblast growth factor (bFGF, 20 ng/mL, MCE). Cultures were maintained at 37°C in a humidified incubator with 5% CO_2_. After 3 days of culture, tumor sphere-formation was assessed.

Tumor spheres were defined as single, compact, and round spheroids with a diameter ≥ 50 μm. Loose aggregates or irregularly shaped cell clusters were excluded from the sphere count. Sphere formation efficiency was calculated as the percentage of wells forming qualified tumor spheres relative to the total number of seeded wells.

### 2.13 Quantitative reverse transcription PCR analysis

Total RNA was extracted using TRIzol reagent (Invitrogen), and reverse transcription was performed with the PrimeScript RT reagent kit (Takara) according to the manufacturer’s instructions. qRT-PCR was conducted using SYBR Green Master Mix (Applied Biosystems) on a QuantStudio 6 Flex system (Thermo Fisher). Relative gene expression was calculated by the 2^-ΔΔCt method, with GAPDH as the internal control. Primers for *OCT4, SOX2, CD133, AKAP12*, and *GAPDH* were synthesized by Sangon Biotech.

### 2.14 Small interfering RNAs transfection

SiRNAs targeting *AKAP12* and corresponding negative control (scramble siRNA) were purchased from GeneAdv. SKOV3 cells were transfected using CALNP RNAi *in vitro* reagent (D-nano Therapeutics) according to the manufacturer’s protocol. Knockdown efficiency was confirmed by qRT-PCR 24 hours post-transfection.

### 2.15 Cell viability assay and IC50 determination

After transfection, SKOV3 cells were seeded into 96-well plates and treated with various concentrations of cisplatin (Sigma-Aldrich) for 48 hours. Cell viability was assessed using the CCK-8 assay (Beyotime). Absorbance at 450 nm was measured using a microplate reader (Bio-Rad). Dose–response curves were generated, and IC50 values were calculated using nonlinear regression analysis in GraphPad Prism 9.0.

### 2.16 Transwell migration assay

Cell migration was evaluated using 24-well Transwell chambers with 8-μm pore size membranes (Corning). After transfection, 5 × 10^4^ SKOV3 cells were suspended in serum-free medium and seeded into the upper chamber, while the lower chamber contained medium supplemented with 10% FBS. After 24 hours of incubation, cells on the upper surface were removed, and migrated cells on the lower surface were fixed with 4% paraformaldehyde, stained with 0.1% crystal violet, and counted in five randomly selected fields under the microscope.

### 2.17 OC organoids

OC organoids were derived from OC tumors via a previously described method (58). Briefly, tumor pieces were minced and enzymatically digested using 1mg/mL collagenase (Solarbio, Beijing, China) and 10 μM Y-27632 (MedChemExpress, NJ, USA) for 45 minutes at 37°C. Established organoid cell lines were expanded by plating organoids with Matrigel Matrix (ECM; Corning, NY 14831, USA) cultured in OC Organoid Kit (Biogenous, Jiangsu, China) at 5% CO_2_, 37°C. Media was replaced every 2–3 days.

### 2.18 Immunohistochemical staining

For immunohistochemical analysis, surgical specimens were processed following standard protocols. Briefly, tissue samples were fixed in 10% formalin, embedded in paraffin, and sectioned at a thickness of 4 micrometers using a microtome. The sections were then mounted on glass slides and dewaxed in xylene followed by graded ethanol washes. Antigen retrieval was performed using a citrate buffer (pH 6.0) in a microwave oven. After blocking endogenous peroxidase activity with 3% hydrogen peroxide and non-specific binding sites with 10% normal serum, the slides were incubated overnight at 4°C with primary antibodies specific to the *AKAP12* or *OX40L* (1:200 dilution). Subsequently, the slides were washed and incubated with appropriate secondary antibodies conjugated to horseradish peroxidase for 1 hour at room temperature. The immune reaction was visualized using 3,3’-diaminobenzidine tetrahydrochloride (DAB) as the chromogen, and the sections were counterstained with hematoxylin. Finally, the slides were dehydrated through graded ethanols, cleared in xylene, and mounted with a permanent mounting medium.

### 2.19 Statistical analysis

Statistical analyses in our study were performed by R studio (version 4.3.1). Analyses of variance for two-sample continuous variables were calculated using t-tests. Nevertheless, for multiple samples and non-normally distributed variables, Wilcox test and Anova analysis could determine whether the difference between groups had statistical significance. All statistical analyses of trials were performed using GraphPad Prism 9.0. Quantitative data are presented as the mean ± standard error of the mean (SEM) from at least three independent experiments. Comparisons between two groups were performed using an unpaired two-tailed Student’s t-test. For drug sensitivity assays (IC50 calculation), nonlinear regression analysis with a log(inhibitor) vs. normalized response curve was applied. Alternatively, *p* < 0.05 was considered to be statistically significant (ns, not significant, *p < 0.05, **p < 0.01, ***p < 0.001, ****p < 0.0001).

## 3 Results

### 3.1 Identification of stemness genes and tumor classification

**Figure 1:**
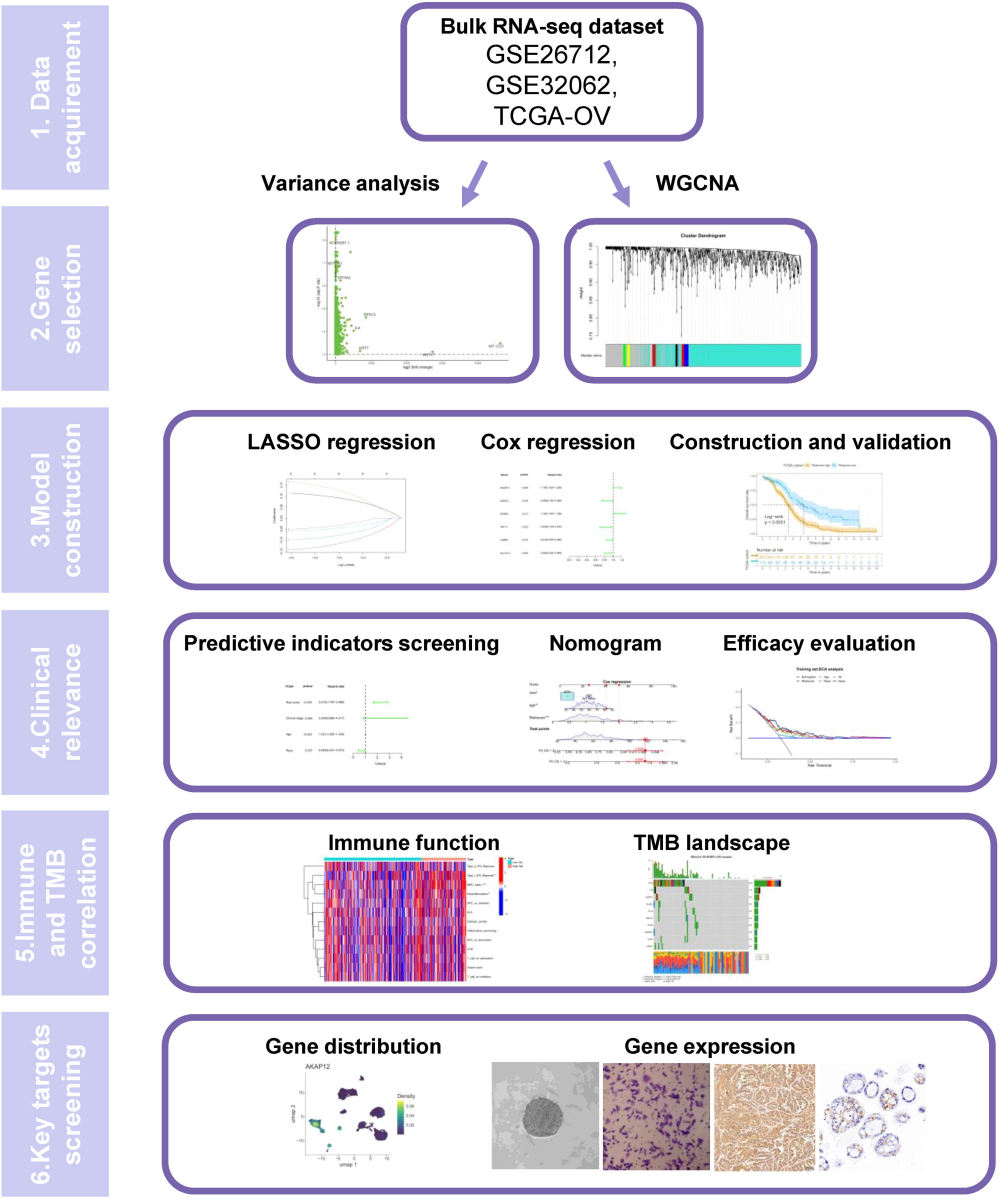
A schematic flow of the whole study.

**Figure 1** depicts the workflow of this study. To select a novel stemness gene set, we extracted the transcriptome sequencing data of SKOV3 cells from the GEO database (GSE232783) and explored related biomarkers that could maintain the dedifferentiation of OC cells by comparing the gene expression profiles of adherent and stem SKOV3 cells. By setting the threshold of logFC > 1 and adjusting *p* < 0.05, we obtained 3066 differentially expressed genes (DEGs) that were highly upgraded in SKOV3 cell stemness. To explore the potential functions of the stemness genes, we conducted Gene Ontology (GO) and Kyoto Encyclopedia of Genes and Genomes (KEGG) enrichment analyses and Gene Set Enrichment Analysis (GSEA). GO enrichment analysis indicated that stemness genes play a key role in the regulation of autophagy, positive regulation of cellular catabolic processes, responses to oxidative stress, protein targeting, and macroautophagy (**Supplementary Figure S1A**). They also serve as regulators of the cell-substrate junction, focal adhesion, and nuclear speck in terms of cellular components. The result of molecular function depicted they were correlated with molecular adaptor activity, cadherin binding, protein-macromolecule adaptor activity and ubiquitin−like protein ligase binding. The results of KEGG enrichment analysis illustrated that stemness genes mainly enriched in amyotrophic lateral sclerosis, human papillomavirus infection, non−alcoholic fatty liver disease, mRNA surveillance pathway, and biosynthesis of amino acids (**Supplementary Figure S1B**). GSEA showed that the expression of stemness genes could lead to the downregulation of the BDP1_TARGET_GENES and ZNF8_TARGET_GENES pathways (**Supplementary Figure S1C**).

**Figure 2:**
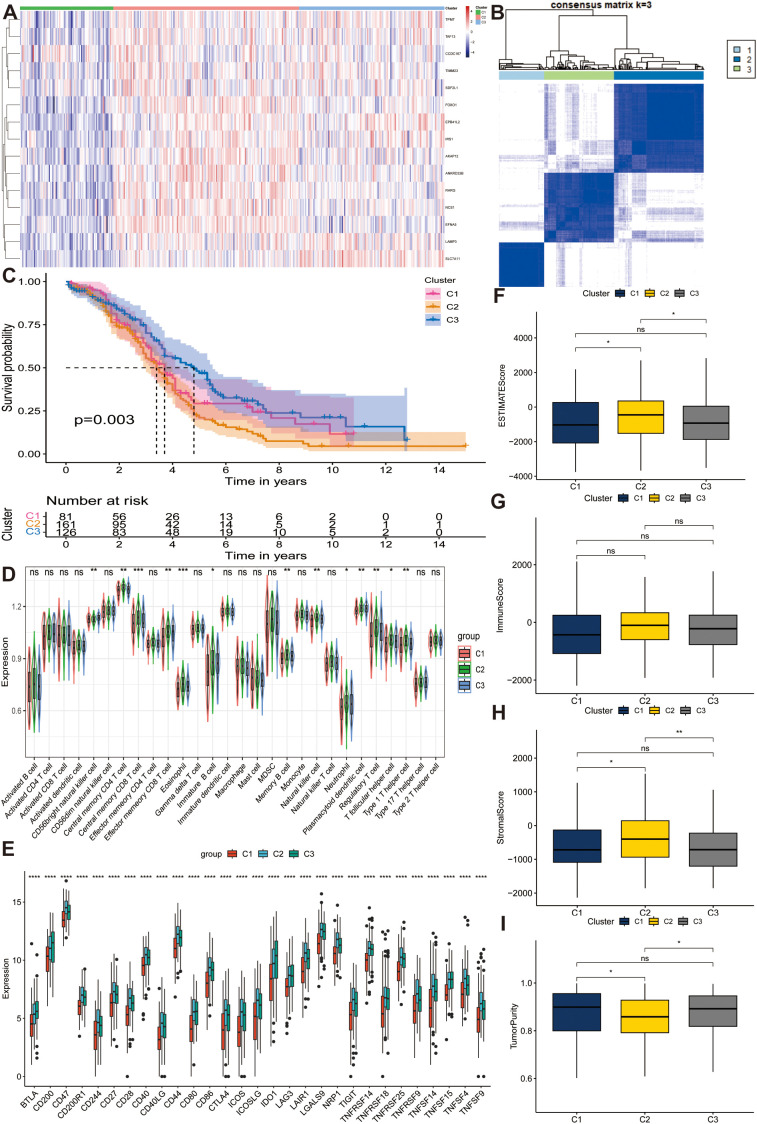
Distinguishing distinct stem subgroups and delineating correlated characteristics in The Cancer Genome Atlas Program (TCGA) cohort. **(A)** The expression profiles of different stem genes in three stem sub clusters. **(B)** The consensus matrix for ovarian cancer (OC) samples when k = 3. **(C)** Kaplan-Meier (KM) curves manifest the survival discrepancy among three groups. **(D, E)** The distribution of diverse immune cells and the expression of immune checkpoints among different groups. **(F–I)** Box plots display the distribution of estimate scores, immune scores, stromal scores and tumor purity among three clusters. ns, not significant, *p < 0.05, **p < 0.01, ***p < 0.001, ****p < 0.0001.

To select favorable stemness genes and assign prognostic significance, we collected 88 normal and 379 OC samples from GTEx and TCGA databases, respectively. After identifying the DEGs between the normal and OC samples, we intersected the results of the two-variance analysis. Subsequently, we conducted a univariate Cox regression analysis and determined 15 prognostic stemness genes (*AKAP12, ANKRD33B, CCDC167, EFNA5, EPB41L2, FOXO1, IRS1, LAMP3, NCS1, RARG, SDF2L1, SLC7A11, TAF13, TIMM23*, and *TPMT*) that were differentially expressed in OC tissues. Consensus clustering analysis was performed to determine whether the stemness phenotype was crucial in tumorigenesis based on 15 prognostic stemness genes. We stratified all patients into k (k = 2-9) different subtypes to determine the overall prognostic value of the genes and the stability of the models under different circumstances. Based on the cumulative distribution function (CDF) curves and the relative change in area under the CDF curves (**Supplementary Figures S2A, B**), we divided all samples into three subtypes, which achieved the best distinctiveness among the subtypes and the heatmap depicts the expression profile of the stemness genes (**Figure 2A, B**). We found that the C2 cluster had the highest stemness phenotype. Moreover, the result of KM analysis also illustrated that cluster 2 had the worst prognosis (*p* < 0.01) (**Figure 2C**).

### 3.2 Exploration of TME and prognostic characteristics in different tumor subtypes

To investigate the influence of stemness genes on tumor samples, we calculated and quantified the abundance of 28 immune cells and the expression of multiple immune checkpoints among the three subtypes. Subsequently, we conducted a Wilcoxon test to further compare the immunological landscapes between the two risk categories. The results indicated that, compared to others, C2 cluster had the highest abundance of central memory CD4^+^ T cells, central memory CD8^+^ T cells, effector memory CD8 + T cells, eosinophils, immature B cells, memory B cells, natural killer cells, neutrophils, plasmacytoid dendritic cells, regulatory T cells, T follicular helper cells, and type 1 T helper cells, whereas CD56 bright natural killer cells were downregulated in this cluster (all *p* < 0.05) (**Figure 2D**). Most immune checkpoints such as CD40, CD44, and CD86 were overexpressed in Cluster 2 (all *p* < 0.05), illustrating the promising effectiveness of immune-targeted therapies for this subtype (**Figure 2E**). Furthermore, we evaluated the TME-related indicators encompassing ESTIMATEScore (**Figure 2F**), ImmuneScore (**Figure 2G**), StromalScore (**Figure 2H**), and TumorPurity (**Figure 2I**) in different subtypes and found that cluster 2 had a high degree of immune infiltration compared to other groups (all *p* < 0.05), which corresponded with the analysis of immune cells, judging from the results of Wilcoxon test. In conclusion, we filtered out a stemness cluster with the worst prognosis. The highest immune infiltration and stemness genes played a key role in the tumorigenesis and advancement of OC.

### 3.3 Screening of hub genes via ssGSEA and WGCNA

**Figure 3:**
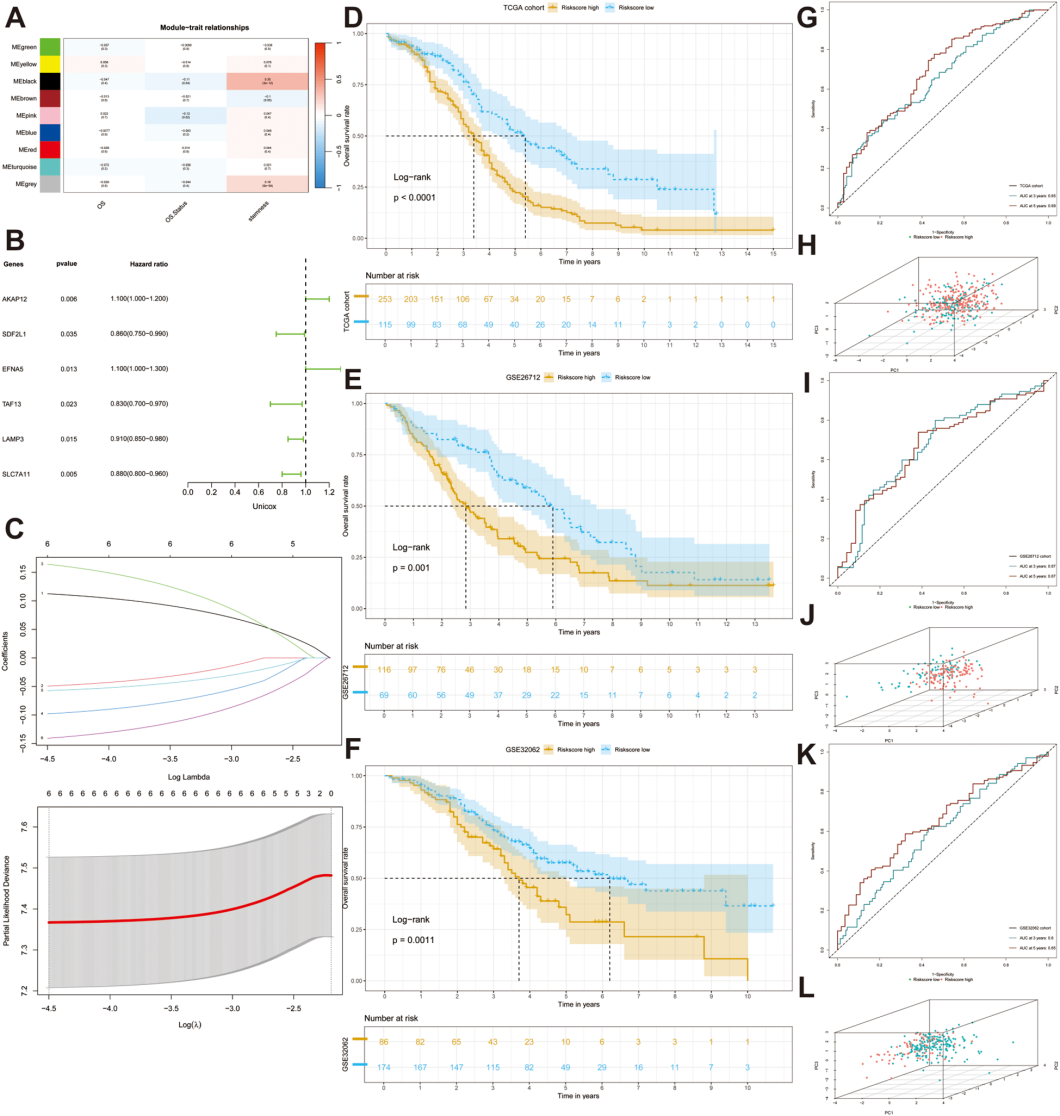
Construction and validation of a stem gene signature. **(A)** A heatmap depicts the correlation and the confidence coefficients between each indicator and nine generated modules utilizing weighted correlation network analysis (WGCNA). **(B)** A forest plot depicts the prognostic value of all stem genes calculated by univariate Cox analysis. **(C)** Selection for six desirable prognostic genes to construct prognostic model based on the optimal parameter λ utilizing Least absolute shrinkage and selection operator (Lasso) Cox regression. **(D–F)** KM analyses are performed in three datasets (TCGA-OV, GSE26712, GSE32062). **(G, I, K)** ROC curves illustrated the sensitivity and specificity of the models. **(H, J, L)** Three-dimensional Principal Component Analysis (PCA) diagrams displaying the distribution of samples.

To determine the degree of stemness in each sample, we calculated the stemness phenotype score for each individual using the ssGSEA algorithm. Subsequently, we conducted WGCNA to identify gene modules related to stemness. When the optimal soft threshold was set to 12, the co-expression network was close to a scale-free network (no scale R^2 = 0.9), and the mean connectivity was stable (**Supplementary Figure S3A**). Finally, genes sharing similar patterns were clustered into the same modules, and genes in TCGA-OV cohort were clustered into nine modules, as indicated by the clustering dendrogram (**Supplementary Figure S3B**) and we extracted 230 genes in the black and gray modules that were strongly correlated with stemness (*p* < 0.001) for further analysis (**Figure 3A**). The network heatmap plot shows the correlations between each module.

### 3.4 Construction and validation of prognostic model based on the hub genes

To explore the adverse effects of stemness genes on patient prognosis, we performed univariate Cox regression analysis to filter out prognostic genes (*p* < 0.05). The forest plot depicted the distribution of the six significant prognostic genes (**Figure 3B**). To prevent overfitting of the model, we conducted LASSO regression analysis to found the optimal λ and encompassed six genes to construct the prognostic model: *AKAP12, EFNA5, LAMP3, SDF2L1, SLC7A11*, and *TAF13* (**Figure 3C**). We then computed the risk score for each sample using the coefficients and expression data of prognostic genes using the following formula: risk score = (*AKAP12* × 0.12620) + (*EFNA5* × 0.19042) - (*LAMP3* × 0.06674) - (*SDF2L1* × 0.05986) - (*SLC7A11* × 0.15868) - (*TAF13* × 0.11205). With the optimal cutoff value serving as the dividing line, we successfully separated all patients into high- and low-risk groups. The KM curve indicated that patients in the high-risk group had a poorer prognosis than those in the other groups (*p* < 0.0001) (**Figure 3D**). ROC curve analysis was performed to evaluate the specificity and accuracy of our model and the 3-year and 5-year AUCs were 0.65 and 0.69, respectively (**Figure 3G**). The three-dimensional PCA plot revealed the distribution of tumor samples and indicated good discrimination between the different risk subgroups (**Figure 3H**).

Subsequently, we acquired the clinical information and transcriptomic sequencing data from 185 patients with OC from the GEO database (GSE26712) to validate the accuracy of the prognostic model. After calculating the risk score, the patients were divided into high- and low-risk groups based on the optimal cut-off value. KM analysis indicated significant survival differences between the two risk subgroups (**Figure 3E**), and AUCs at 3 and 5 years were 0.67 and 0.67, respectively, which confirmed the accuracy of the model (**Figure 3I**). Principal component analysis (PCA) demonstrated that the two groups showed favorable differentiation (**Figure 3J**). Moreover, a satisfying discrepancy in the OS between two risk-groups was also observed in an additional dataset (GSE32062) comprising 260 patients (**Figures 3F, L**), reinforcing the robustness of our findings. 3-year and 5-year AUCs were 0.60 and 0.65, respectively (**Figure 3K**).

### 3.5 Establishment and evaluation of a nomogram

**Figure 4:**
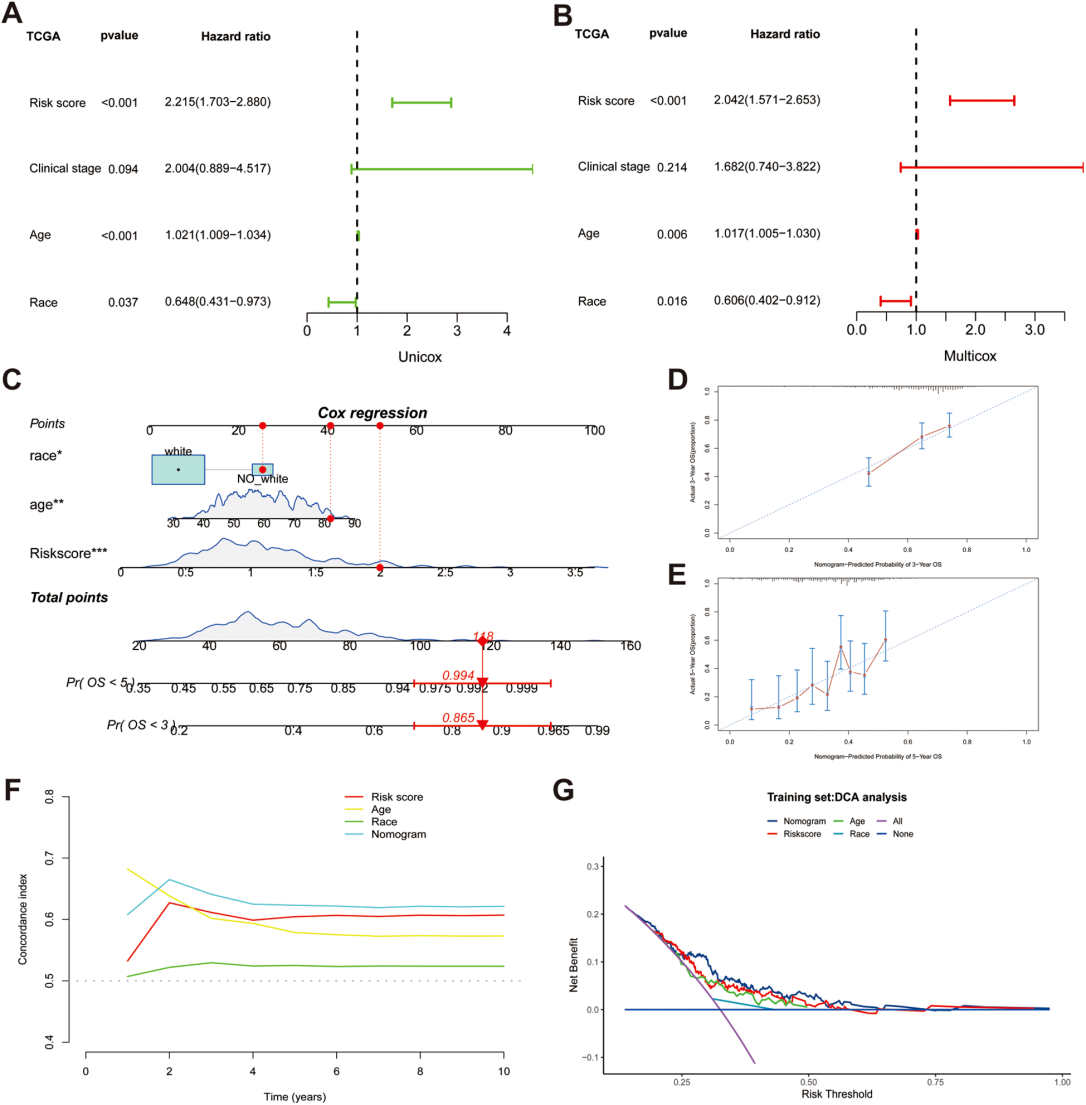
Establishing a nomogram based on risk score and clinical variables for predicting the 3-year and 5-year survival probability in TCGA cohort. **(A, B)** Univariable and multiple Cox regression for identifying independent predictive factors. **(C)** A nomogram for predicting overall survival (OS). **(D, E)** Calibration plots for predicting 3-year and 5-year OS. **(F)** Comparing the concordance indexes (C-indexes) of nomogram, risk score, race and age. **(G)** Distribution Curve Analysis (DCA) analysis of diverse indicators.

To identify independent prognostic factors, we employed univariate and multivariate Cox regression analyses and found that age (HR = 1.021, *p* < 0.001), race (HR = 0.648, *p* < 0.05), and risk score (HR = 2.215, *p* < 0.001) all had prognostic significance (**Figures 4A, B**). Based on three independent indicators, a nomogram was generated to predict the 3-year and 5-year survival probabilities of patients in the TCGA cohort (**Figure 4C**). Furthermore, we utilized a calibration curve to evaluate the accuracy of prediction. The predictive and actual curves almost coincided, indicating the high efficacy of the nomogram (**Figures 4D, E**). In addition, the highest C-index demonstrated the robust predictive power of the nomogram compared with other clinicopathological parameters (**Figure 4F**). DCA revealed that the nomogram and risk score had the best net benefit compared with other clinicopathological parameters, and the nomogram encompassing prognostic indicators achieved the best effectiveness according to the curve (**Figure 4G**).

### 3.6 Investigation of TME, TMB and drug sensitivity

**Figure 5:**
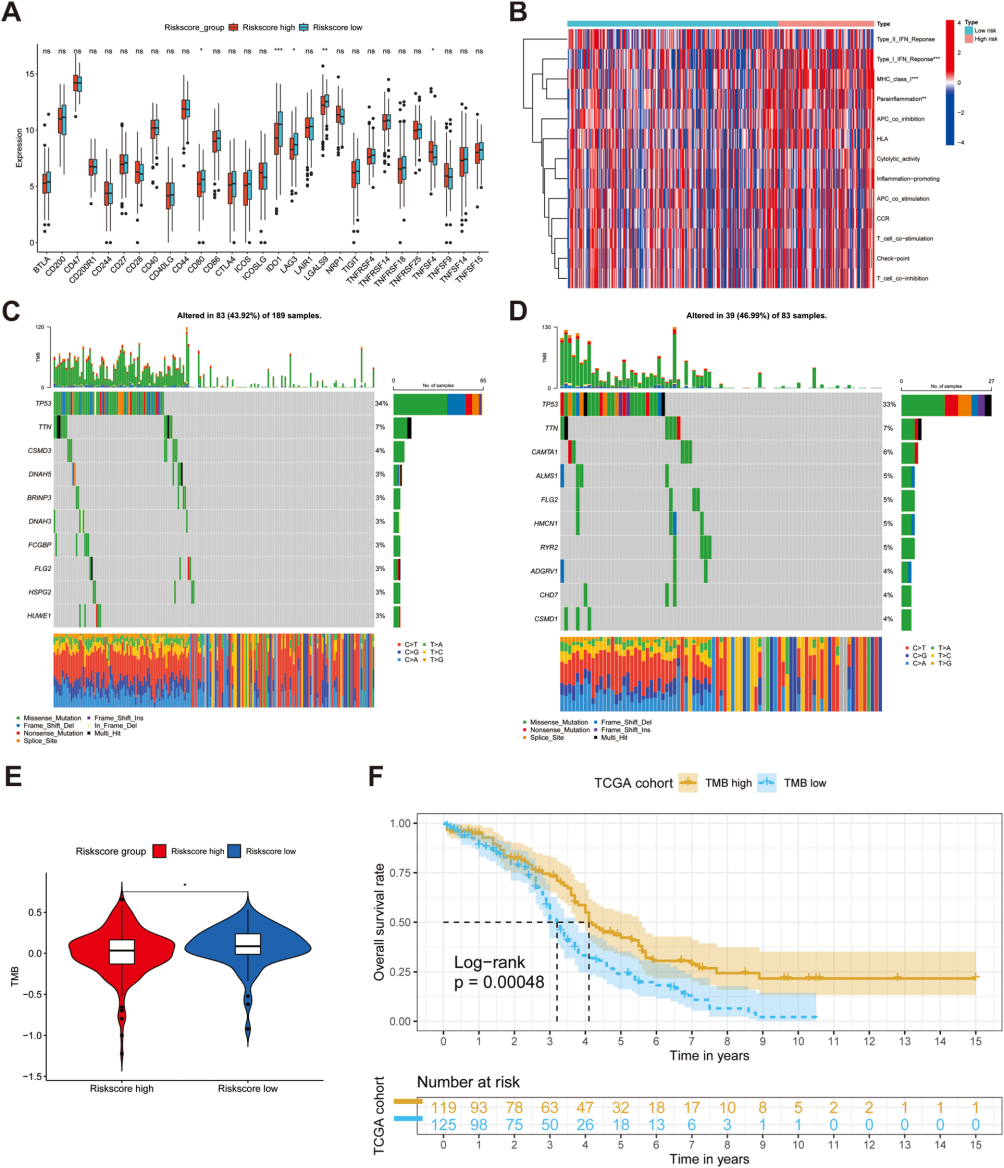
Investigating the difference in terms of tumor microenvironment (TME) between high- and low-groups. **(A, B)** The difference of the expression of immune checkpoints and immune functions between two groups. **(C, D)** Waterfall diagrams reveal the landscape of TMB in high-risk (N = 189) and low-risk (N = 83) groups. **(E)** A box plot suggests high-risk group had lower mutations. **(F)** KM analysis demonstrates the different OS between two risk groups. ns, not significant, *p < 0.05, **p < 0.01, ***p < 0.001.

To ascertain the statistical significance of discrepancies between the two risk sub-groups regarding the aspects of TME, TMB and drug susceptibility, a t-test analysis was conducted. We firstly calculated the abundance of 28 immune cells using the ssGSEA algorithm, as mentioned above, and discovered that there was no difference between the high- and low-risk groups in terms of immune infiltration (all *p* > 0.05) (**Supplementary Figure S4A**). Additionally, we compared the expression of immune checkpoints between the two risk subgroups and found that *CD80, IDO1, LAG3*, and *LGALS9* were significantly downregulated in the high-risk group (all *p* < 0.05) (**Figure 5A**). Notably, while the expression of *TNFSF4*, also known as *OX40L*, was upregulated in the high-risk population, whereas the expression of its receptor, *TNFRSF4* (*OX40*), did not concurrently show an increase. To reveal the potential immune function, we conducted an analysis of immune pathways, and the results demonstrated the activation of Type I IFN Response, MHC class I, and para-inflammatory pathways (all *p* < 0.01) (**Figure 5B**). Waterfall plots show the landscape of the mutation rates of the top ten genes’ mutation rate between the high- and low-risk groups (**Figures 5C, D**). These results reveal that risk genes can increase the mortality rate by activating these potential pathways. The box plot results indicated that the low-risk group had higher TMB, which corresponded to the waterfall plot (**Figure 5E**). After evaluating the TMB in patients with OC, we separated all patients into high or low TMB groups with the threshold of the median TMB, and KM analysis showed that lower TMB was linked to worse prognosis (*p* < 0.001) (**Figure 5F**). Therefore, we hypothesized that poor outcomes may be related to drug tolerance. Finally, we meticulously performed a sensitivity evaluation of some immune checkpoint inhibitors and chemotherapeutic drugs and found higher IC50 values for Acetalax, Erlotinib, Rapamycin, and Savolitinib in the high-risk group, revealing an increase in drug resistance (all *p* < 0.05) (**Supplementary Figure S4B**).

### 3.7 Establishment of a single-cell landscape for OC

**Figure 6:**
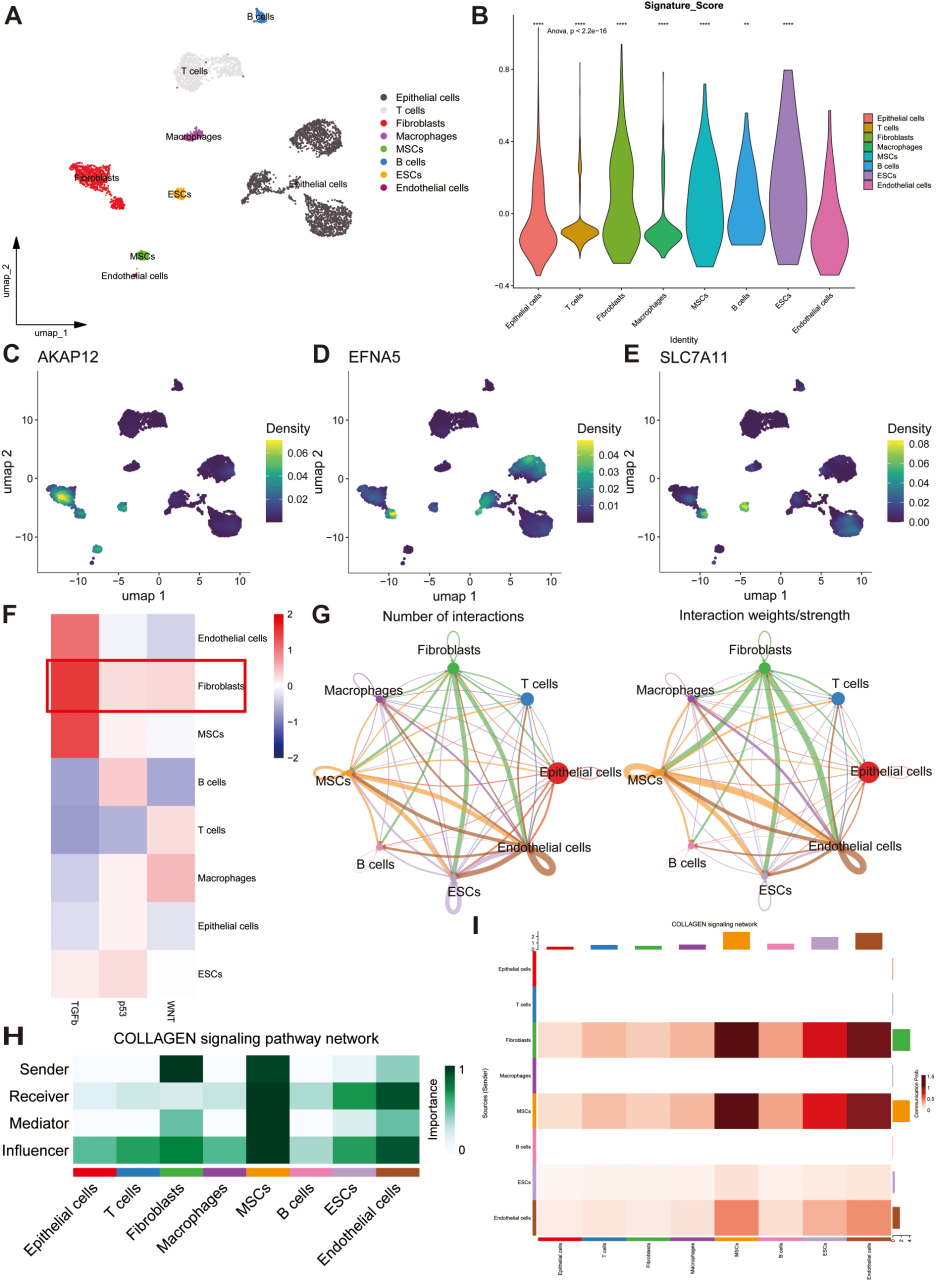
Single-cell analysis reveals the potential mechanisms of stem genes in remodeling TME. **(A)** The overall single-cell landscape of ovarian samples depicted by the uniform manifold approximation and projection (UMAP). **(B)** Distributions of stemness signature score in all cell types depicting by violin plot. **(C–E)** The enrichment extent of *AKAP12*, *EFNA5* and *SLC7A11* in all cell types. **(F)** The strength and activation of stem signals among different cell clusters. **(G)** Cell-cell communication analysis illustrating the interactions and weights of inter-cellular signals. **(H)** A heatmap showing the potential roles of all cell types in COLLAGEN signaling pathway. **(I)** The strength of outgoing and incoming signaling patterns among all cell types manifested by a heatmap. ns, not significant, **p < 0.01, ****p < 0.0001.

To characterize the distribution of prognostic genes at the single-cell level, we extracted scRNA-seq data from five primary OC tissues encompassing two high-grade OC samples and three low-grade OC samples from the GEO database (GSE235931). Under stringent quality control, the employment of UMAP culminated in the categorization of 4773 cells and 27753 features into 11 discrete clusters. According to the cell markers described in previous articles, we separated all cells into eight clusters: epithelial cells, T cells, fibroblasts, macrophages, mesenchymal stem cells (MSCs), B cells, Embryonic Stem Cells (ESCs), and endothelial cells (**Figure 6A**). The UMAP map revealed the abundance of distinct cells derived from multiple samples (**Supplementary Figure S5A**). The heatmap shows the expression of marker genes and top ten expressed genes that represent each cell population (**Supplementary Figures S5B, C**). Meanwhile, to determine the stem content of the TME for OC, we uncovered the significant upregulation of gene signature in fibroblasts by conducting Anova analysis (*p* < 0.001) (**Figure 6B**) and investigated the distribution of three model genes with the largest coefficients (*AKAP12, EFNA5*, and *SLC7A11*) in diverse cell clusters depicted by UMAP maps (**Figures 6C–E**). Furthermore, the signal enrichment analyses illustrated the remarkable expression of stem signals, consisting of TGF-β, p53, Wnt signals (**Figure 6F**), demonstrating that fibroblasts should account for the origin of stemness.

### 3.8 Identification of potential signaling pathways via cell-cell communication analysis

CellChat networks can vividly delineate the weights and numbers of interactions to reveal potential correlations among all cell types. Based on these results, we discovered a strong correlation between fibroblasts and other cell populations (**Figure 6G**). A heatmap was utilized to reflect the strength of the top 46 intricate cell-cell signaling pathways, and the *COLLAGEN*, *LAMININ*, *APP*, *FN1*, and *VISFATIN* signaling pathways were found to be crucial for tumorigenesis (**Supplementary Figure S5D**). By calculating the centrality scores of the *COLLAGEN* signaling pathway, we identified that fibroblasts mainly function as senders, whereas endothelial cells serve as receivers and influencers. Alternatively, MSCs may play a significant role in the regulation of all intercellular signaling pathways (**Figures 6H, I**). Overall, these prognostic genes may induce OC development by regulating the proliferation of endothelial cells, MSCs, and fibroblasts.

### 3.9 *AKAP12* promotes stemness phenotypes and is associated with immune checkpoint *OX40L* expression in OC

**Figure 7:**
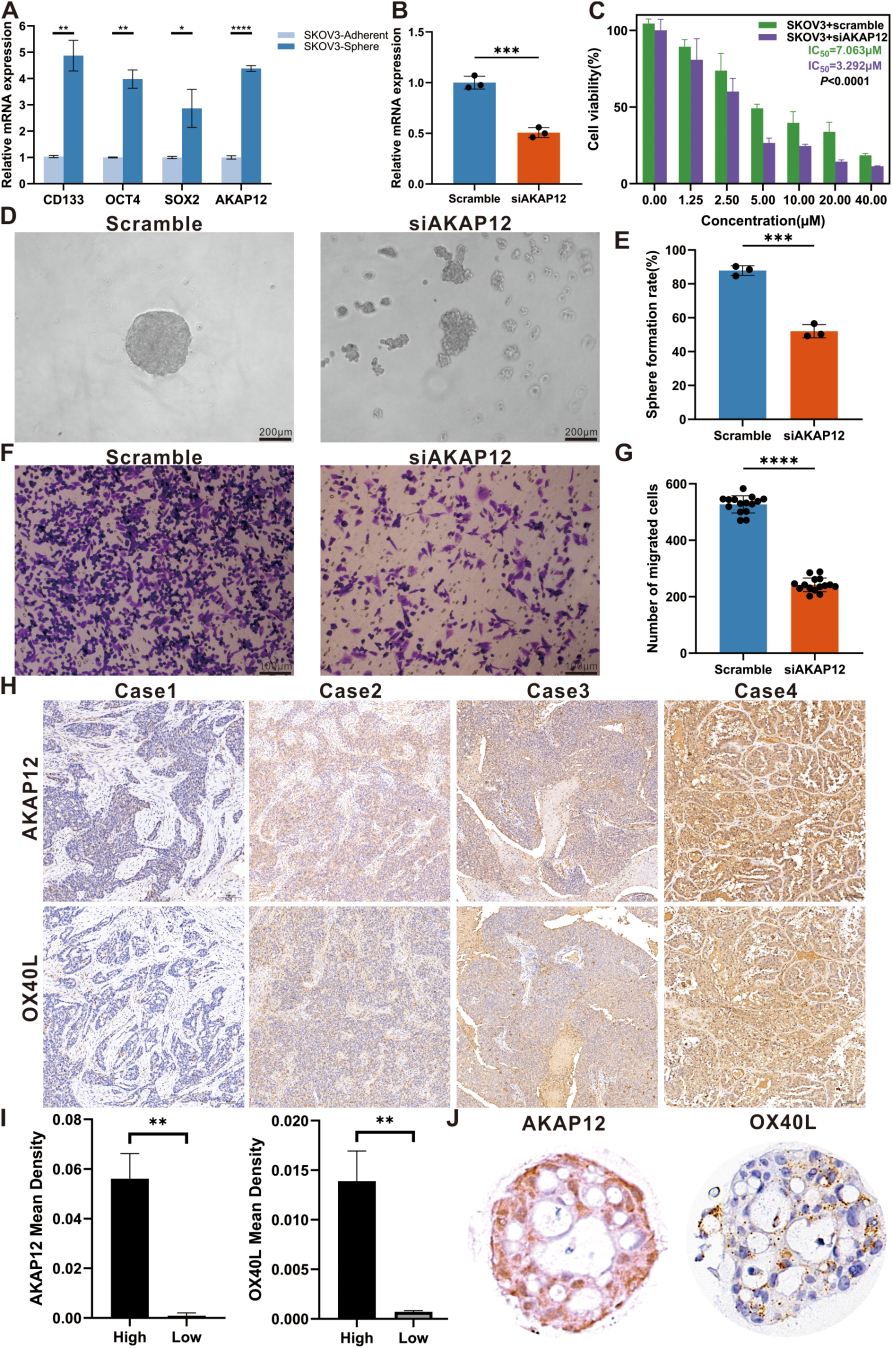
Effects of *AKAP12* knockdown on stemness, migration, and chemosensitivity in ovarian cancer cells, and expression analysis in clinical samples and organoids. **(A)** Relative expression levels of classical stemness markers (*OCT4*, *SOX2*, *CD133*) and *AKAP12* in SKOV3 cells cultured under adherent versus tumor sphere-forming conditions, assessed by qRT-PCR. (n = 3). **(B)** Verification of *AKAP12* knockdown efficiency in SKOV3 cells by qRT-PCR. (n = 3). **(C)** Cell viability curves and IC50 values for cisplatin in scramble- and si*AKAP12*-transfected SKOV3 cells, assessed by CCK-8 assay. (n = 3). **(D)** Representative tumor sphere images showing the morphological differences between scramble and si*AKAP12*-transfected SKOV3 cells after 3 days of culture. **(E)** Quantitative analysis of sphere formation efficiency in scramble and si*AKAP12* groups. (n = 3). **(F)** Representative Transwell images showing the migrated SKOV3 cells after *AKAP12* knockdown compared to scramble controls. (n = 3). **(G)** Quantification of migrated cell numbers per field in scramble and si*AKAP12*-transfected SKOV3 cells. (n = 3). **(H)** Representative immunohistochemical images showing differential expression of AKAP12 and OX40L in four ovarian cancer tissue samples. **(I)** Quantitative comparison of staining intensity between high- and low-expression subgroups. **(J)** Expression levels of AKAP12 and OX40L in ovarian cancer organoids. Data are presented as mean ± SEM. *p < 0.05, **p < 0.01, ***p < 0.001, ****p < 0.0001.

Considering both the model coefficients and the affinity of the antibodies, we meticulously appraised the actual expression of *AKAP12*, which obtained the highest coefficient and robustly represented the extent of stemness in multiple OC samples. To further elucidate the role of *AKAP12* in OC, a series of functional and expression analyses were performed. Firstly, qRT-PCR analysis demonstrated that the expression levels of classical stemness markers [*OCT4* (59), *SOX2* (60), and *CD133* (61)] were significantly elevated in SKOV3 cells cultured under tumor sphere-forming conditions compared with adherent cultures. Importantly, *AKAP12* expression was simultaneously upregulated alongside these stemness-associated genes, suggesting a close association between *AKAP12* expression and the stemness phenotype in OC cells (**Figure 7A**). Furthermore, siRNA-mediated knockdown of *AKAP12* in SKOV3 cells effectively reduced its mRNA expression, confirming the knockdown efficiency (**Figure 7B**). CCK-8 assays revealed that *AKAP12* knockdown enhanced the sensitivity of SKOV3 cells to cisplatin, as evidenced by a decreased IC50 value compared with scramble controls (**Figure 7C**). Functionally, *AKAP12* knockdown markedly impaired tumor sphere-formation. Representative images showed that si*AKAP12*-transfected SKOV3 cells failed to form compact, round spheroids compared with the scramble group (**Figure 7D**), and quantitative analysis demonstrated a significant reduction in sphere formation efficiency (**Figure 7E**). Moreover, Transwell migration assays indicated that the migratory ability of SKOV3 cells was significantly suppressed following *AKAP12* knockdown, with fewer migrated cells observed both qualitatively (**Figure 7F**) and quantitatively (**Figure 7G**).

Although previous studies have seldom explored the relationship between *AKAP12* expression and clinicopathological parameters, our study addressed this gap through immunohistochemical analysis of OC tissues and organoid models. Representative images of *AKAP12* and *OX40L* expression in clinical samples and cultured organoids are shown in **Figure 7H**. Based on the calculated mean staining density, samples were stratified into high- and low-density groups. A positive correlation between *AKAP12* and *OX40L* expression was observed using t-test analysis (**Figure 7I**), and this association was further confirmed in OC organoids (**Figure 7J**).

These findings suggest that *AKAP12* serves as a key regulator of stemness and malignant phenotypes in OC and may represent a promising prognostic indicator. Targeting *AKAP12* could potentially reduce stemness and drug resistance, while enhancing the therapeutic efficacy of *OX40* agonists in patients with high *AKAP12* expression.

## 4 Discussion

OC is a type of cancer with high heterogeneity and is traditionally stratified according to its degree of differentiation. Clinicians tend to neglect the histological differentiation of OC and fail to select appropriate treatment targets (62). Previous studies have suggested that OC stemness characteristics are strongly related to metastasis and chemotherapy resistance (15). Under these circumstances, establishing novel stemness gene sets to evaluate the degree of stemness of tissues from patients with OC and exploring individualized immunotherapy treatments is advised.

A previous study confirmed the significant role of aggresomes in the contribution and maintenance of OC stemness and identified DEGs by comparing the gene expression of OC cells before and after differentiation (34). Further functional enrichment analysis of DEGs revealed that stemness genes mainly activate autophagy, adhesion, and protein adaptor activity signaling pathways to maintain OC dedifferentiation. Studies have reported that Enhanced autophagic flux is observed in OC stem cells, and autophagy can induce the progression of OC through *FOXA2* (63). Furthermore, researchers have identified a significant function for cell adhesion molecules, which mediate intracellular adhesion and induce the dispersion of OC cell spheroids to contribute to metastasis (64). The discovery of this study corresponded with the functional enrichment analysis. GSEA demonstrated that the *BDP1* and *ZNF8* signaling pathways were downregulated throughout progression. Accumulating evidence indicates that both *BDP1* and *ZNF8* can serve as biomarkers for the diagnosis of OC (65, 66).

Among the three stemness clusters subgrouped by the consensus clustering approach based on 15 prognostic stemness genes, C2 cluster had a highly enriched degree with the worst prognosis. Having calculated the immune infiltration of the three subtypes, we discovered that C2 subtype had a favorable degree of immune infiltration, whereas it had the lowest tumor purity compared to the others. Additionally, high stromal scores are correlated with high-risk and unfavorable prognosis (46, 67, 68). After identifying C2 as the distinguishing subtype, we explored the distribution of immune cells. The results indicated that the abundance of most memory cells, regulatory cells, and helper cells significantly increased in the C2 subtype, and the proportion of CD56dim nature killer cells was lower than that in the other subtypes. Evidence has demonstrated that the activation of regulatory and memory cells could suppress the immune response in anti-tumor processes, and they accumulate in the malignant ascites fluid together with epithelial cells, which could contribute to poor prognosis (69). Nevertheless, CD56dim nature killer cells are enriched in OC-related ascites and elicit anti-tumor cytotoxicity (70). Moreover, immune checkpoint expression demonstrated that C2 subtype is suitable for immunotherapy.

To further elucidate the effect of stemness characteristics on prognosis, we employed WGCNA and selected genes in the black and grey modules, which were highly related to stemness, for survival analysis. Using univariate Cox regression analysis and LASSO Cox regression, we successfully constructed a prognostic model comprising six stemness-related genes (*AKAP12, EFNA5, LAMP3, SDF2L1, SLC7A11*, and *TAF13*), two of which (*AKAP12* and *EFNA5*) showed great relevance to stemness and poor prognosis with the largest coefficients. The expression profile of *AKAP12* elevated in paclitaxel- and platinum-resistant serous OC cells and correlates with poor OS and progression-free survival (PFS) (71). High-grade serous carcinoma tends to overexpress *EFNA5*, especially at aggressive stages. *EFNA5* inhibits tumor-suppressive signaling pathways, leading to tumorigenesis and drug resistance (72). Through delineating distinct stem cell characteristics, a previous study (73) also defined a prognostic signature comprising nine stem genes (*SFRP2*, *MFAP4*, *CCDC80*, *COL16A1*, *DUSP1*, *VSTM2L*, *TGFBI*, *PXDN*, and *GAS1*) that are upregulated in high-risk group and elucidated potential mechanisms, through which these risk factors may promote the proliferation and metastasis of OC cells.

To further highlight the differences between the models constructed in our and those in other studies, we conducted an additional assessment of their predictive performance using bioinformatics analysis. Specifically, we calculated the risk score of all patients based on the expression and coefficients of prognostic genes, and the risk score also reacted to stem abundance. KM analysis substantiated the distinguishing function of the model in the training and validation cohorts. Meanwhile, the ROC curves confirmed the robustness of predicting 3- and 5-year OS. In comparison with the aforementioned model, our model exhibited superior predictive capacity, as evidenced by 3-year and 5-year ROC values of 0.65 and 0.69 in the TCGA cohort, and exhibiting 0.67 and 0.67 in the GSE26712 cohort, 0.60 and 0.65 in the GSE32062 cohort, respectively. Conversely, the ROC values reported in the other study were 0.626 and 0.671 in one cohort, and 0.622 and 0.583 in the other cohort for the same time frames.

After confirming that the risk score was an independent factor by performing univariate and multivariate Cox regression analyses, we established a nomogram encompassing all risk factors to predict 3-year and 5-year survival rates. In alignment with the methodologies employed in the previous study, we also utilized calibration curves to demonstrate the predictive efficacy of our nomogram. Moreover, the calculation of C-index, collaborating with the development of DCA curves underscored the model’s effectiveness and its substantial net clinical benefit.

To clarify the comprehensive landscape of TME, the abundance of distinct immune cell populations and the activity of multiple immune pathways between two risk subgroups were investigated. Intriguingly, through no substantial differences regarding the distribution of immune cells was observed, the notable upregulation of regulatory T cells (Tregs) captured our attention. Tregs, comprising a minor subset of CD4^+^ T cells, play a pivotal role in sustaining self-tolerance of the immune system via modulating the proliferation and differentiation of effector cells, not only in the context of cancer (74), but also in inflammatory diseases (75, 76). Additionally, prior research (77) has established that the expression of Foxp3, a marker specifically associated with Tregs, is regulated by p53. Drawing upon the conclusion from other literatures (78–80), which suggested that p53 enhances the chemotherapy toleration and promotes the generation of cancer stem cells by modulating cancer cell quiescence or activating multiple signaling pathways, it’s reasonable for us to hypothesize that cancer cells exhibiting a high level of stemness may demonstrate pronounced expression of p53 and in turn can facilitate the proliferation of Tregs. Conversely, the highly immunosuppressive TME, influenced by Tregs, may also contribute to the emergence and growth of cancer stem cells.

On the other aspect, the collaborative upregulation of the content of type 1 helper cells (Th1 cells) and IFN-γ was observed across groups ranging from low to high stemness. IFN-γ, predominately secreted by Th1 cells, induces inflammatory response and stimulates antitumor immune processes by triggering diverse downstream signaling pathways (81). cGAS/STING, as the major downstream pathway, upregulate a variety of immune checkpoints, implying the potential efficacy of applying immune checkpoint inhibitors in populations with high stem extent (82, 83).

Based on the results of immune checkpoint expression, we hypothesized that an imbalance between OX40 and OX40L expression could hinder the activation of NK and cytotoxic T cells (84, 85). Utilizing anti-OX40 immunostimulants may further enhance therapeutic efficacy for patients with high levels of stemness and reduce the incidence of resistance to platinum (86, 87). TMB analysis demonstrated that low TMB may be related to poor clinical outcomes. This phenomenon is caused by decreased sensitivity to immunotherapy in a population with a high TMB (88). Finally, a drug sensitivity analysis indicated that the application of an *OX40* immunity activator could provide new insights into the treatment of OC. The results of IHC staining also revealed the viability of the application of *OX40* agonists in OC sections with high levels of stemness, which has not yet been recognized by clinicians, and could provide novel insights for the therapy of patients with recurrent refractory OC. Tumor organoids, a novel *in vitro* model, are derived from tumor tissues and can simulate tumor proliferation in the host and respond to external chemotherapy or immune drugs (89–91). By employing this simulation methodology, we reinforced the healing efficacy of *OX40* agonists in drug-resistant and highly stemmed populations. Referring to a previous study (92), the combined application of *OX40* and IFN-γ with STING agonists may achieve satisfactory anti-tumor efficacy.

Single-cell transcriptomics can improve the ability to describe cellular states (93). By integrating multiple single-cell datasets, we successfully revealed the distribution of various cells in the TME encompassing OC samples at the high and low stages. Moreover, the main stemness genes (*AKAP12*, *EFNA5*, and *SLC7A11*) were enriched in fibroblasts, ESCs, and MSCs, implying that these cell types drive stem cell production. Previous studies have shown that cancer-associated fibroblasts and MSCs promote the invasiveness, stemness, and metastasis of OC (94, 95). Cell-cell interactions and signaling pathway analyses have attached great importance to the role of fibroblasts, MSCs, and endothelial cells in the COLLAGEN signaling pathway. PLOD enzymes, which have implications for cancer aggressiveness, can promote collagen cross-linking and increase the firmness of the tumor matrix (96). In addition, cancer-related fibroblasts maintain OC stem cell growth by activating the Wnt5a signaling pathway (97). Under these circumstances, fibroblasts, serving as senders, could induce the proliferation of MSCs and endothelial cells and contribute to the formation of OC stem cells. Ultimately, the inducing role of *AKAP12* in stem phenotype of OC was verified *in vitro* experiments and clinical samples.

Our study had some limitations. First, single-cell and transcriptome data were acquired from public databases, and increase in the sample size is required to improve the scientific validity of the findings. Although we confirmed a positive relationship between the expression of *OX40* and immune checkpoints in clinical samples, cytological experiments should be considered to validate the relevant biological functions. Overall, the model that we established could evaluate the actual stem extent for patients with OC and guide efficient therapeutic strategies to improve their prognosis.

## 5 Conclusion

In summary, we identified a novel stemness gene set by investigating differential change in pre- and post- dedifferentiation OC cells. Utilizing an unsupervised clustering algorithm, we successfully validated the favorable performance of the gene set in distinguishing stemness clusters, and discerned a malignant cluster with a high degree of stemness and poor prognosis. Owning to the potential prognostic value of stemness genes, we established a risk model to enhance prognostic prediction and guide personalized treatment. Having discussed the correlation between the risk score, TME and drug resistance, we performed a creatively discovery, that advised clinicians to activate *OX40*/*OX40L* checkpoints to reduce the occurrence of drug resistance and improve patients’ prognosis. Single-cell analysis revealed the potential mechanism driving stem cell production.

## Data Availability

The original contributions presented in the study are included in the article/**Supplementary Material**. Further inquiries can be directed to the corresponding authors.

## Supplementary material

Supplementary Figure 1 The functional enrichment analysis of differentially expressed genes (DEGs). The plots depict the enrichment results of Gene Ontology (GO) **(A)**, Kyoto Encyclopedia of Gene and Genome (KEGG) **(B)** enrichment analysis and Gene Set Enrichment Analysis (GSEA) **(C)** of DEGs. BP, Biological process; MF, Molecular function; CC, Cellular components.

Supplementary Figure 2 The correlated performance of different clustering results. **(A)** Cumulative distribution function (CDF) curves revealing the probability distribution of different subtype numbers (k =2-9). **(B)** The relative change of area under Delta CDF curve.

Supplementary Figure 3 The generation process of correlated modules via WGCNA. **(A)** The diagrams manifest the scale independence and the mean connectivity of multiple soft-thresholding power values. **(B)** A cluster dendrogram constructed by the weighted correlation coefficients. Genes with similar expression pattern were clustering into the same module. Each color represents a module and each branch represents a gene.

Supplementary Figure 4 The landscape of TME and drug susceptibility in two risk subgroups. **(A)** The distribution content of 28 immune cells. **(B)** The IC50 values of common chemotherapy agents are calculated in the high- and low-risk groups. ns, not significant, **p* < 0.05, ***p* < 0.01.

Supplementary Figure 5 The single-cell atlas of TME consisting of high- and low-grade OC samples. **(A)** The comparation of cells distribution in primary OC samples depicted by UMAP plots. Heatmaps manifesting the expression of annotation genes **(B)** and the top ten genes **(C)**. **(D)** A heatmap revealing the strength of outgoing and incoming signaling patterns among all cell types.
