# Supplementary figures and images for: Stemness-driven clusters in ovarian cancer: immune characteristics and prognostic implications
Source: Front Oncol. 2025 Jun 11;15:1577283. doi: 10.3389/fonc.2025.1577283 (PMC12187856; doi:10.3389/fonc.2025.1577283)

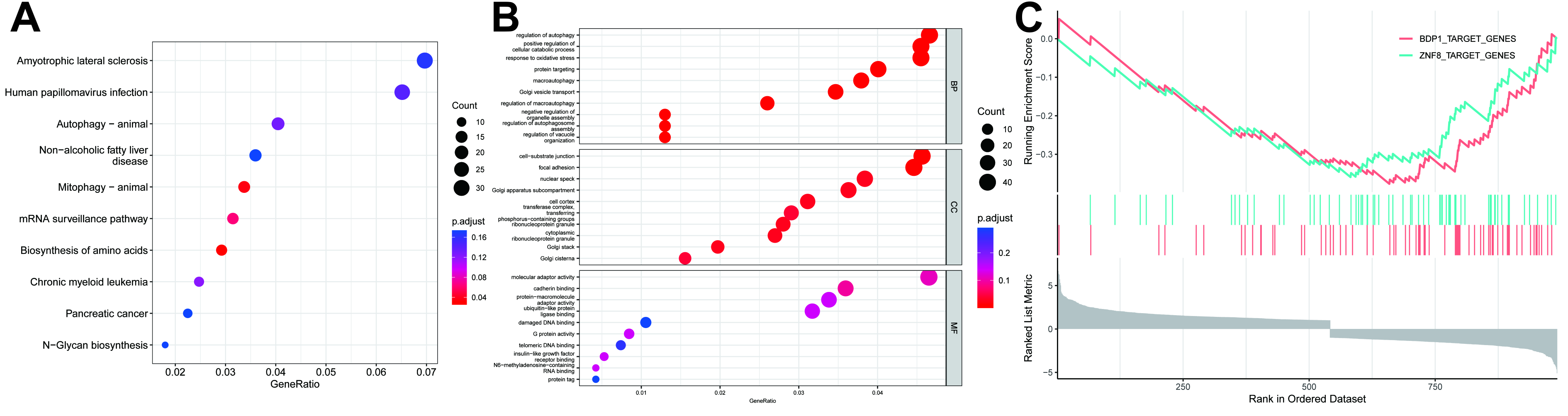

Supplement: Supplementary Figure 1 — The functional enrichment analysis of differentially expressed genes (DEGs). The plots depict the enrichment results of Gene Ontology (GO) (A), Kyoto Encyclopedia of Gene and Genome (KEGG) (B) enrichment analysis and Gene Set Enrichment Analysis (GSEA) (C) of DEGs. BP, Biological process; MF, Molecular function; CC, Cellular components. [file Image1.tif]

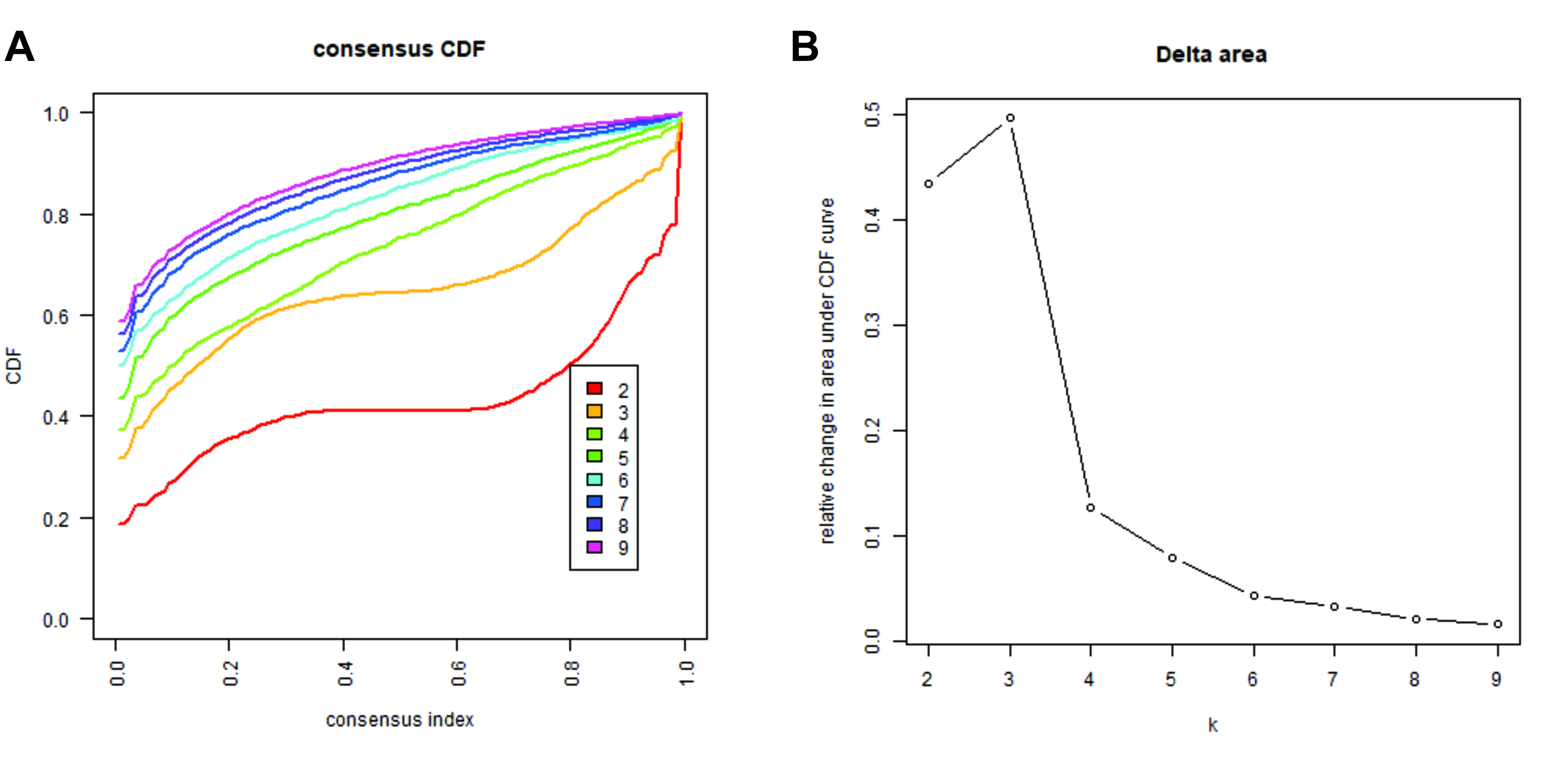

Supplement: Supplementary Figure 2 — The correlated performance of different clustering results. (A) Cumulative distribution function (CDF) curves revealing the probability distribution of different subtype numbers (k =2-9). (B) The relative change of area under Delta CDF curve. [file Image2.tif]

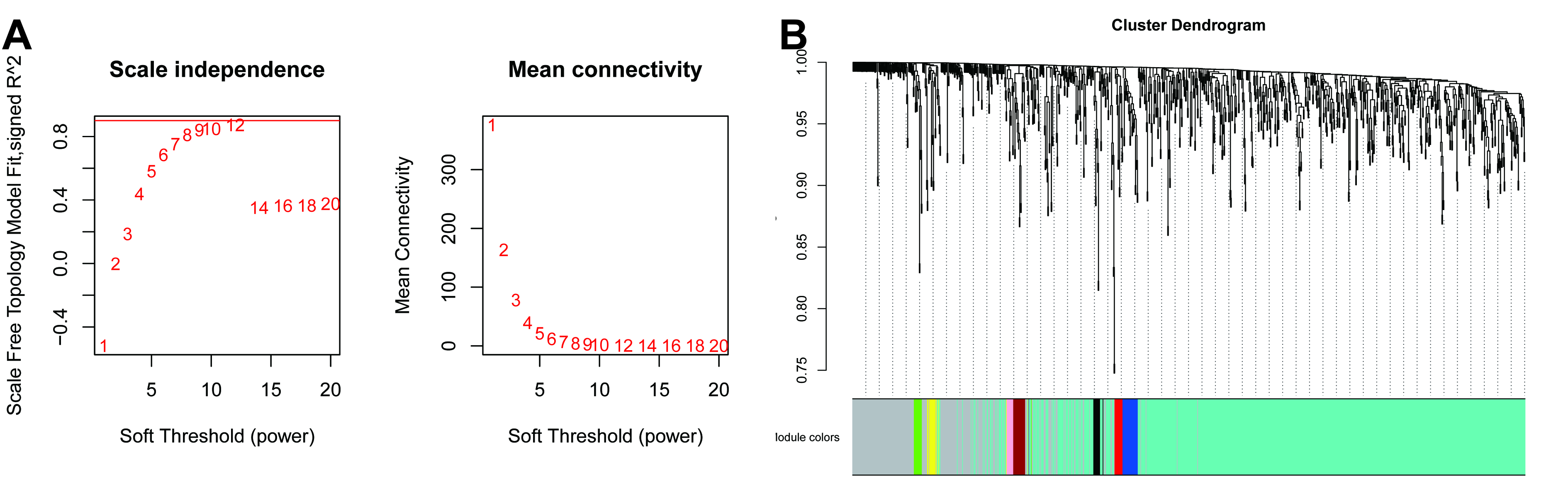

Supplement: Supplementary Figure 3 — The generation process of correlated modules via WGCNA. (A) The diagrams manifest the scale independence and the mean connectivity of multiple soft-thresholding power values. (B) A cluster dendrogram constructed by the weighted correlation coefficients. Genes with similar expression pattern were clustering into the same module. Each color represents a module and each branch represents a gene. [file Image3.tif]

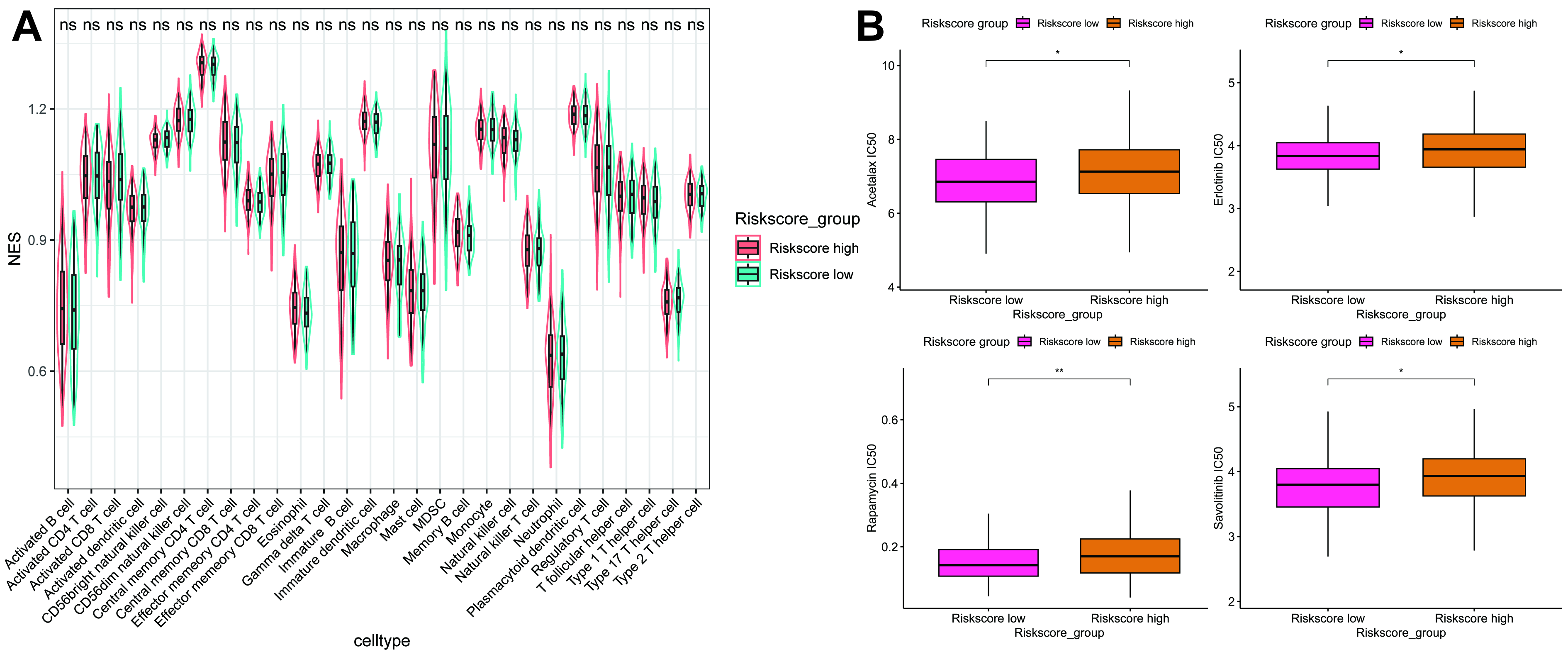

Supplement: Supplementary Figure 4 — The landscape of TME and drug susceptibility in two risk subgroups. (A) The distribution content of 28 immune cells. (B) The IC50 values of common chemotherapy agents are calculated in the high- and low-risk groups. ns, not significant, *p < 0.05, **p < 0.01. [file Image4.tif]

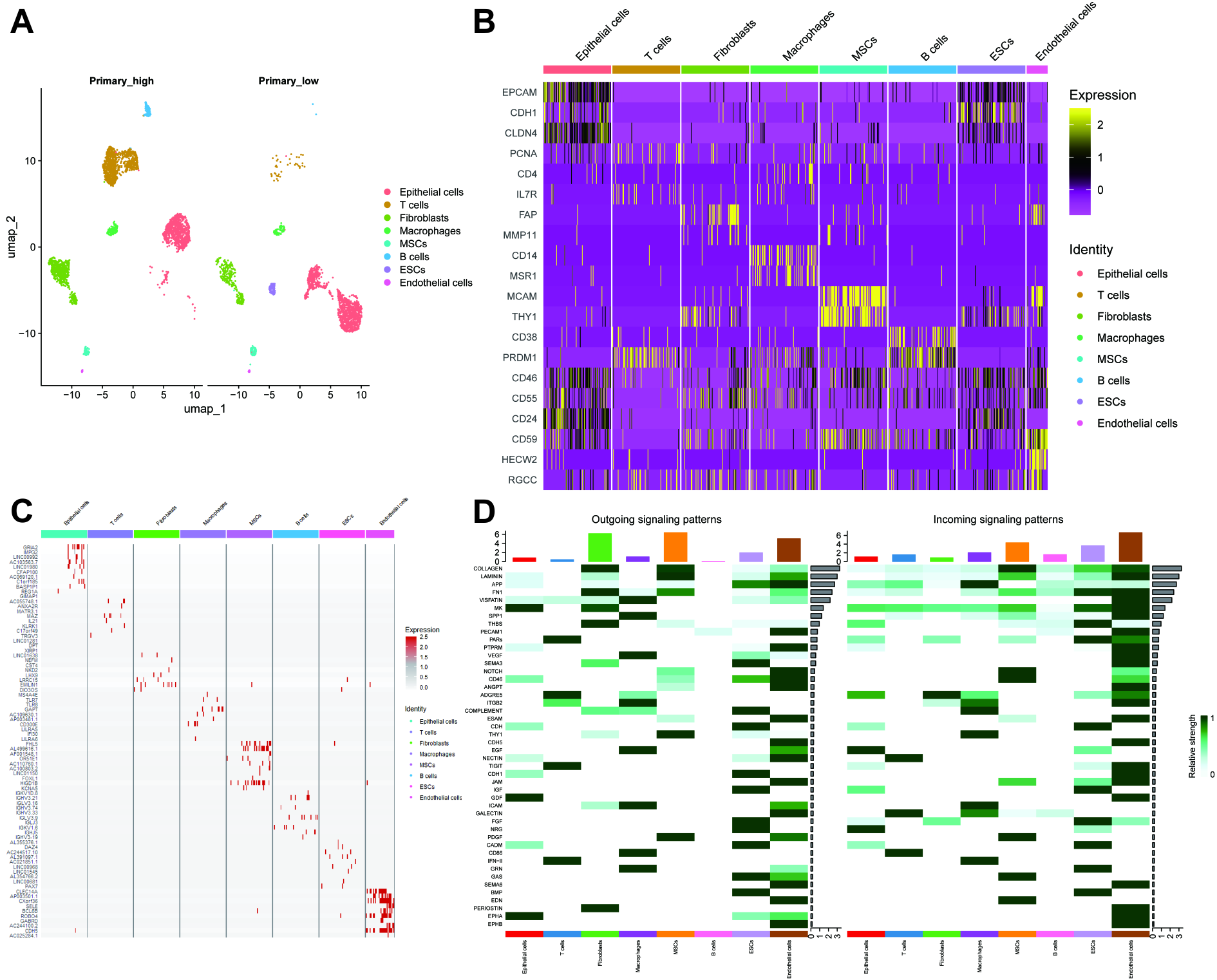

Supplement: Supplementary Figure 5 — The single-cell atlas of TME consisting of high- and low-grade OC samples. (A) The comparation of cells distribution in primary OC samples depicted by UMAP plots. Heatmaps manifesting the expression of annotation genes (B) and the top ten genes (C). (D) A heatmap revealing the strength of outgoing and incoming signaling patterns among all cell types. [file Image5.tif]
